# Plants grown in Apollo lunar regolith present stress-associated transcriptomes that inform prospects for lunar exploration

**DOI:** 10.1038/s42003-022-03334-8

**Published:** 2022-05-12

**Authors:** Anna-Lisa Paul, Stephen M. Elardo, Robert Ferl

**Affiliations:** 1 Interdisciplinary Center for Biotechnology Research and Horticultural Sciences Department, University of Florida, Gainesville, FL USA; 2 Department of Geological Sciences, University of Florida, Gainesville, FL USA; 3 UF Research and Horticultural Sciences Department, University of Florida, Gainesville, FL USA

**Keywords:** Abiotic, Transcriptomics, Plant molecular biology

## Abstract

The extent to which plants can enhance human life support on other worlds depends on the ability of plants to thrive in extraterrestrial environments using in-situ resources. Using samples from Apollo 11, 12, and 17, we show that the terrestrial plant *Arabidopsis thaliana* germinates and grows in diverse lunar regoliths. However, our results show that growth is challenging; the lunar regolith plants were slow to develop and many showed severe stress morphologies. Moreover, all plants grown in lunar soils differentially expressed genes indicating ionic stresses, similar to plant reactions to salt, metal and reactive oxygen species. Therefore, although in situ lunar regoliths can be useful for plant production in lunar habitats, they are not benign substrates. The interaction between plants and lunar regolith will need to be further elucidated, and likely mitigated, to best enable efficient use of lunar regolith for life support within lunar stations.

## Abstract (web summary)

*Arabidopsis* plants were seeded onto lunar soil samples taken directly from the Apollo 11, 12, and 17 missions. Transcriptomic analyses reveal that plants grown in lunar soil differentially express genes associated with salt, metal, and ROS stress.

## Introduction

The return of humans to the Moon during the NASA Artemis program^1^ has elevated scientific interest in the lunar environment and its impact on terrestrial biology^2,3^. Since the return to the Moon is envisioned as a dedicated, longer-term commitment to lunar exploration, questions of the lunar environmental impact on biology and biological systems have become a significant part of the lunar exploration agenda. Plants have long been envisioned as part of lunar habitats^4–8^ and exploration environments^9,10^. However, until the current study, the interactions between lunar materials and terrestrial biology were unaddressed in the era of modern molecular biology, and there had yet to be any experiments where plants were actually grown in the true lunar regolith. Understanding the impact of sustained exposure of terrestrial biology to lunar regolith, and determining the efficacy of lunar regolith as a viable in situ resource, is important to the concept of returning to the Moon for long durations. Therefore, we used plant growth and gene expression (e.g., ^11–13^) to both tests the fundamental impact of lunar regolith on terrestrial biology, and provide an initial evaluation of regolith as a matrix for plant growth systems in lunar exploration habitats.

Plants are key components of the biological sciences within and in support of space exploration. As model organisms, plants provide insights into gravity, radiation, and other space-related biological phenomena, and therefore help drive an understanding of the physiological adaptation of terrestrial biology to space exploration environments. There is an expanding wealth of literature documenting this progress, e.g., ^11,14–19^. Plants also make available potentially essential components to the long-term habitation of space and extraterrestrial surfaces by providing food and oxygen, recycling water, and scrubbing carbon dioxide from human habitats. However, many designs of extraterrestrial plant growth systems largely rely on hydroponic systems^20,21^, in part due to the lack of information on the efficacy of using in situ materials, such as planetary regolith, as a growth substrate. Information on plant growth in lunar regolith, therefore, informs fundamental biology interactions within the lunar environment, and helps scale options for the use of plants in lunar life support scenarios.

The Apollo Moon landings were a critical point in the science of space exploration, not only in the accomplishment of lunar visits, but also in that terrestrial biology would be in contact with extraterrestrial material, and samples of that material would be intentionally returned to Earth. The first contact between terrestrial biology and lunar regolith was an unprecedented concern for protecting both the astronauts visiting the Moon as well as the entire Earth biosphere from the potentially harmful effects of returned lunar materials. Novel, extraterrestrial pathogenic microorganisms were the main concern, in addition to toxic mineral compositions and abrasive physical characteristics. To protect the returning human explorers and the terrestrial biosphere, the astronauts and attending medical staff were isolated for 2 weeks upon the return of the early Apollo crews (e.g., ^22^). In addition, lunar samples were intensively isolated in specialized facilities at the Johnson Space Center Lunar Receiving Laboratory^23,24^, where the samples were handled in gloveboxes roughly equivalent to current high-level biological containment. The primary goal was to search for pathogens, and those search procedures relied on direct, though largely transient contact between lunar materials and biology. In the specific case of plants, lunar samples were rubbed onto leaf surfaces and sprinkled onto seedlings and growth media^25^, but plants were never grown in lunar regolith as the support matrix^25–27^. This gap in knowledge is addressed in the following experiment.

**Fig. 1:**
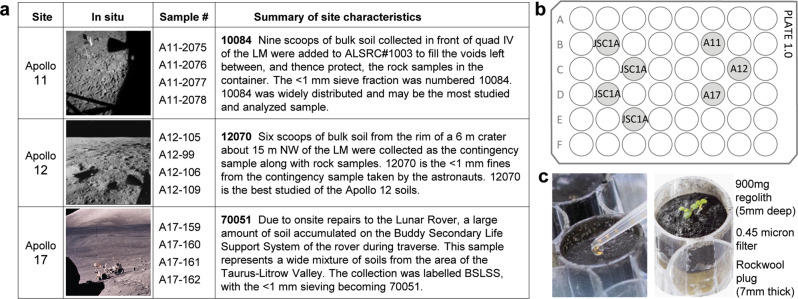
Lunar regolith sources and experiment set up. **a** A summary table of lunar sample sources^50^. **b** We used four replicate plates, each 48-well cell culture plate contained four wells of JSC-1A simulant and three of lunar regolith (one well each per plate) – Apollo 11 (A11), Apollo 12 (A12), Apollo 17 (A17). **c** Each well contained 900 mg of regolith material layered over a 0.45-micron filter and Rockwool wick and was sown with 3-5 Arabidopsis seeds suspended in water (also Supplementary Fig. 1). Lunar In Situ images are publicly available from NASA archives.

We asked the two-tiered question of whether plants can develop successfully in lunar regolith, and if so, what metabolic strategies, as suggested by differential transcriptomes, were utilized to physiologically adapt to growth in this novel environment. Arabidopsis was seeded directly onto samples from Apollo 11 (10084), 12 (12070), and 17 (70051) (Fig. 1a), which represent diverse regolith types. The Apollo 11 and 12 samples are mature and submature regoliths, respectively, due to their relatively long exposure to the lunar surface, whereas the Apollo 17 sample is an immature, shorter surface exposure regolith mixture from multiple areas at the Apollo 17 site^28–31^. All are from areas of the Moon dominated by basaltic mineralogy^28–31^. The lunar simulant JSC-1A^29,32,33^ was used for the terrestrial control material. All lunar regolith samples, as well as the JSC-1A simulant used for controls, were samples sieved to <1 mm particle size.

There area few notable mineralogical and compositional differences between the lunar regoliths and JSC-1A. The lunar samples used here contain 30–52 vol. % agglutinates, which are aggregates containing mineral fragments, nanophase metallic Fe, trapped gases, and glass that form due to micrometeorite impacts^28,29^. Agglutinate and nanophase Fe abundance both increases with regolith maturity as prolonged surface exposure to the solar wind reduces particle size, the increases the conversion rate of grains of Fe metal to nanophase Fe^28,29^. Many regolith simulants such as JSC-1A contain abundant natural volcanic glass, e.g., ^29^, but this material does not fully replicate agglutinate assemblages or morphologies. Additionally, the overall Fe oxidation state of the lunar samples differs from that of JSC-1A. Although to our knowledge, Fe oxidation state ratios have not been quantified in these samples, lunar regolith ubiquitously contains nanophase metallic Fe (partially within agglutinates) in addition to solely Fe^2+^ in mafic silicates and oxides^34^, whereas JSC-1A is known to contain Fe^3+^-bearing Fe-Ti oxides (namely magnetite and chromite) in addition to some proportion of Fe^3+^ in its silicates and glass. Lastly, two of the lunar regoliths (10084 and 70051) are derived from high-Ti basaltic bedrock and have TiO_2_ abundances of 5–7 wt.%, which is considerably higher than that of JSC-1A at 1.85 wt.%, e.g., ^29^.

## Results and discussion

### Plant growth in three sources of lunar regolith

To provide insights into the potential for using lunar regolith to grow plants as part of the Artemis lunar exploration science program and for future sustained lunar habitation, we developed a small-scale system based on 48-well laboratory plates capable of assessing plant growth in small, obtainable amounts of lunar regolith samples returned from the Apollo missions 11, 12, and 17 (Fig. 1a, b and Supplementary Fig. 1). Wild-type Columbia-0 (Col-0) seeds were sown directly on the surface of the lunar regolith (Fig. 1c), and developing roots grew in full contact throughout the volume of the lunar regolith. 900 mg aliquots of the three different lunar regoliths, as well as the JSC-1A lunar simulant, were arranged in four, 48-well plates (Fig. 1b) modified with a subsurface irrigation system (Fig. 1c and Supplementary Fig. 1). After sowing with Arabidopsis seed (Fig. 1c, Supplementary Fig. 1H), the plates in their individual watering trays were transferred to clear, ventilated terrarium boxes (Supplementary Fig. 1I), and placed under growth lights in a secured plant growth room. Growing the plants in the ventilated terrarium boxes reduced airflow, yet simulated an open laboratory environment potentially similar to a human-occupied lunar habitat, as opposed to a sterile growth chamber.

**Fig. 2:**
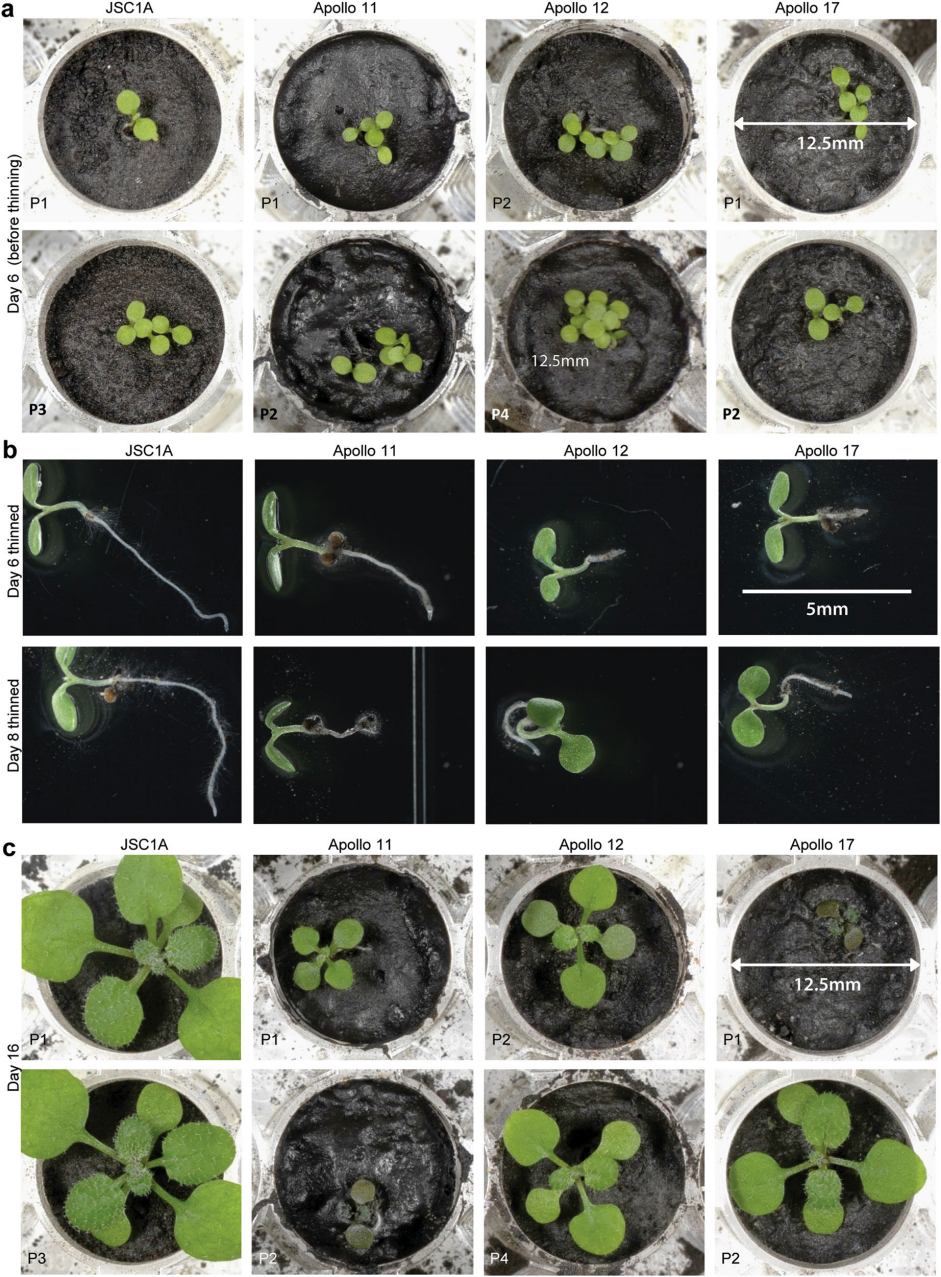
Germination and development in the lunar regolith. **a** Germination rates were close to 100% in all sources of Apollo lunar regolith and indistinguishable from rates in JSC-1A simulant. Two representative wells for JSC-1A and each Apollo site are shown. **b** The seedlings thinned from each well on day 6 or 8 indicated that root growth in lunar regolith is not as robust as in JSC-1A. **c** While germination was uniform among controls and lunar sites, the lunar regolith-grown seedlings did not thrive as compared to the JSC-1A controls. The diameter of the culture plate wells is 12.5 mm (scale bar provided in **c**). All microscope images in **b** are shown to the same scale (scale bar shown in the Apollo 17 image).

**Fig. 3:**
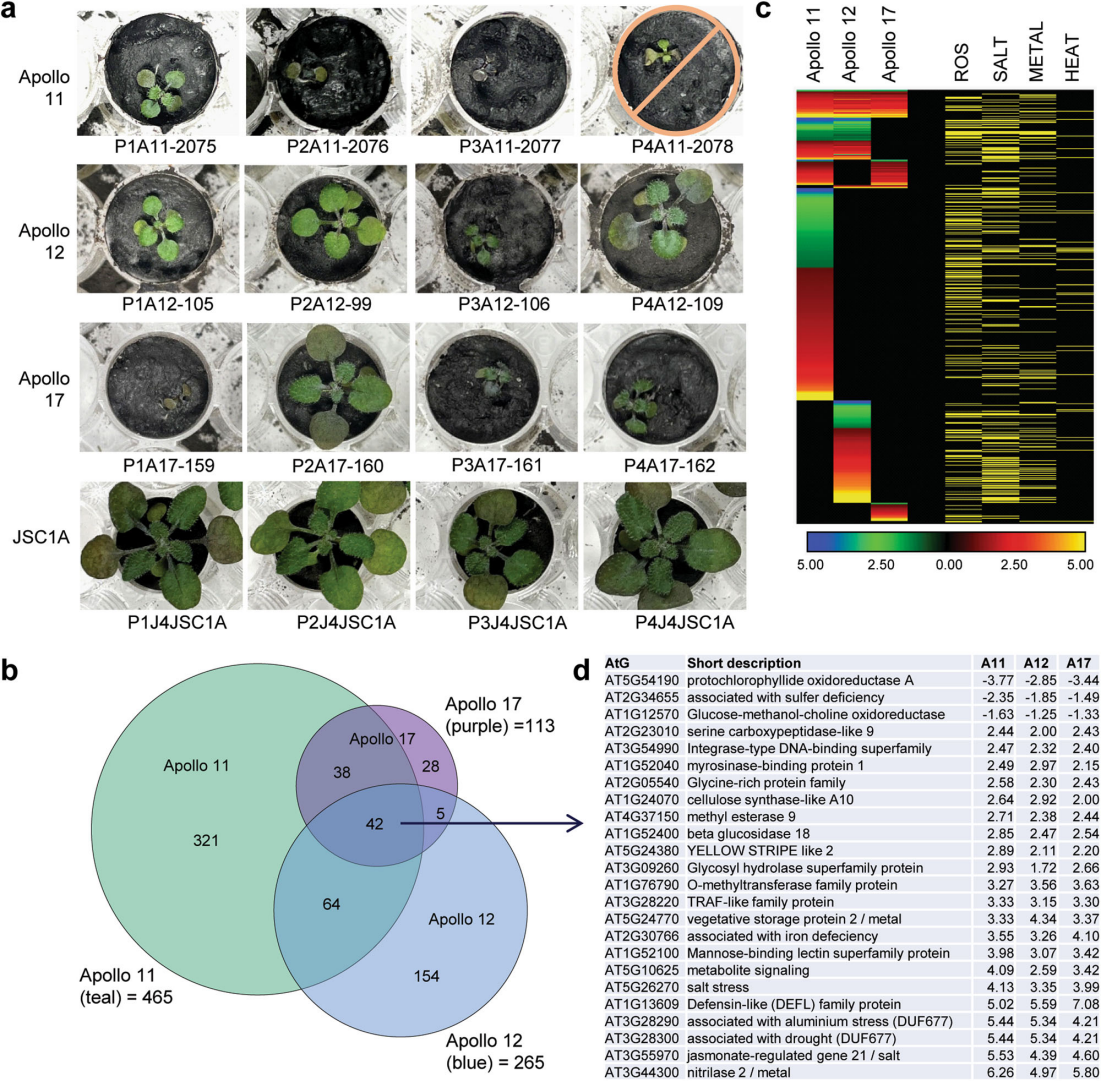
Transcriptome analyses of plants grown in lunar regolith grouped by Apollo site. **a** Three (Apollo 11) to four (Apollo 12, 17) plants from each site comprised the analyses. One Apollo 11 plant did not survive and was excluded from the DEG analysis. The photographs were taken just prior to harvest. **b** Venn diagrams showing the DEG overlap for plants from each site: teal for Apollo 11, blue for Apollo 12, and purple for Apollo 17. **c** Heat maps show the Log2 fold-change of DEGs in plants from each site. Genes associated with ROS, salt, metal, and heat responses are indicated by yellow rows. These associations were primarily derived from the stress transcriptomes and data sets presented in References (^37–40^) and NCBI gene annotations (https://www.ncbi.nlm.nih.gov/gene/823555). **d** 25 of the most highly differentially expressed genes common to all Apollo sites with fold-change presented as Log2. The fully annotated heat map (3 C) is provided as Supplementary Data 1, and the complete, fully annotated list of 3D is provided in Supplementary Data 3).

Germination readily occurred on all samples between 48 and 60 hours after planting, and all lunar seedlings exhibited normal stems and cotyledons (Fig. 2a), indicating that nothing derived from the full contact with the hydrated regolith interfered with the complex set of signaling events required for early aerial development. Between days 6 and 8, each of the plantings was thinned to leave a single plant per well. The roots of the plants thinned from lunar samples were stunted compared to the plants thinned from JSC-1A (Fig. 2b), indicating relative inhibition of root growth in lunar regolith. Aerial growth and development beyond 8 days became slower and more variable in the lunar samples compared to JSC-1A (Fig. 2c). Although there was variability among the individual plant replicates for each of the lunar regolith sites, there were lunar site-specific trends in the development of the plants (Fig. 3a). The rate of development for all plants was monitored daily, and the expansion of the leaf canopy was quantified from top-view photographs (Supplementary Fig. 4). There was almost no variability in the growth rates or morphology among the sixteen JSC-1A replicates (Supplementary Fig. 2). Compared to the JSC-1A replicates, all lunar plants took longer to develop expanded leaves, were smaller in rosette diameter over time, and some were severely stunted and deeply pigmented, a typical indicator of plant stress. Only a few plants developed nearly as well on lunar regolith as those on JSC-1A (Figs. 2b, 3a, Supplementary Fig. 2). The JSC-1A plants maintained a consistently higher rate of growth than plants grown on any of the lunar regolith samples, and the plants grown on Apollo 11 regolith fared worse than plants grown on regolith from Apollo 12 and 17 (Fig. 3 and Supplementary Fig. 4).

### Plant transcriptomes differentiate by Apollo site

To examine the potential bases of the stress morphologies of lunar regolith-grown plants, transcriptome analyses were performed on the entire aerial portions (leaves and small stem) of the plants after 20 days. The gene expression data were parsed based on lunar sample site replicates compared to the JSC-1A controls (Fig. 3, Supplementary Data 1). The plants grown in Apollo 11 regolith showed the greatest number of significantly differentially expressed genes (DEGs) (465), followed by Apollo 12 (265), and Apollo 17 (113) (Fig. 3b). All lunar samples, irrespective of Apollo site, significantly evoked DEGs indicative of a strong stress response, with 71% of the DEGs typically associated with salt, metal, and reactive oxygen species (ROS) stresses^35–39^ (Fig. 3c). The DEGs that were coordinately expressed in all three lunar sites also included a strong representation (29%) of genes associated with nutrient metabolism (Fig. 3d). The most highly repressed and induced (respectively) of the coordinately expressed genes were Protochlorophyllide Oxidoreductase-A (AT5G54190) and a gene of unknown function (AT5G26270), which are both associated with phosphate starvation^40^. Other highly induced genes common to all sites included the well-characterized defense genes Nitilase-2 (AT3G44300, also induced by cadmium and metal ions)^39^, Jasmonate-regulated gene-21 (AT3G55970), and Defensin-like (AT1G13609), plus two genes encoding proteins of unknown function (AT3G28290 and AT3G28300) that are also involved in aluminum toxicity^41^ and jasmonate signaling^42,43^. In addition to the coordinate responses, plants from each of the Apollo samples differentially expressed genes unique to each sample location (Fig. 3c), suggesting a discernable and distinguishing plant response based on lunar soil sample; however, all coordinated and site unique categories indicated a stress response by plants to lunar regolith.

The statistically supported differentiation of transcriptome responses based on lunar sites indicated that plant responses vary based on the lunar regolith source, and supported the overall conclusion that lunar regolith is more stressful than JSC-1A simulant. However, the various growth morphologies demonstrated within the replicates of each lunar regolith sample suggested the potential for a range of success states for lunar plant growth. To investigate the responses of potential growth success states, the transcriptomic data were regrouped based on plant size as one measure of growth success (see Supplementary Fig. 4 for plant growth data).

### Plant transcriptomes differentiate by morphology

**Fig. 4:**
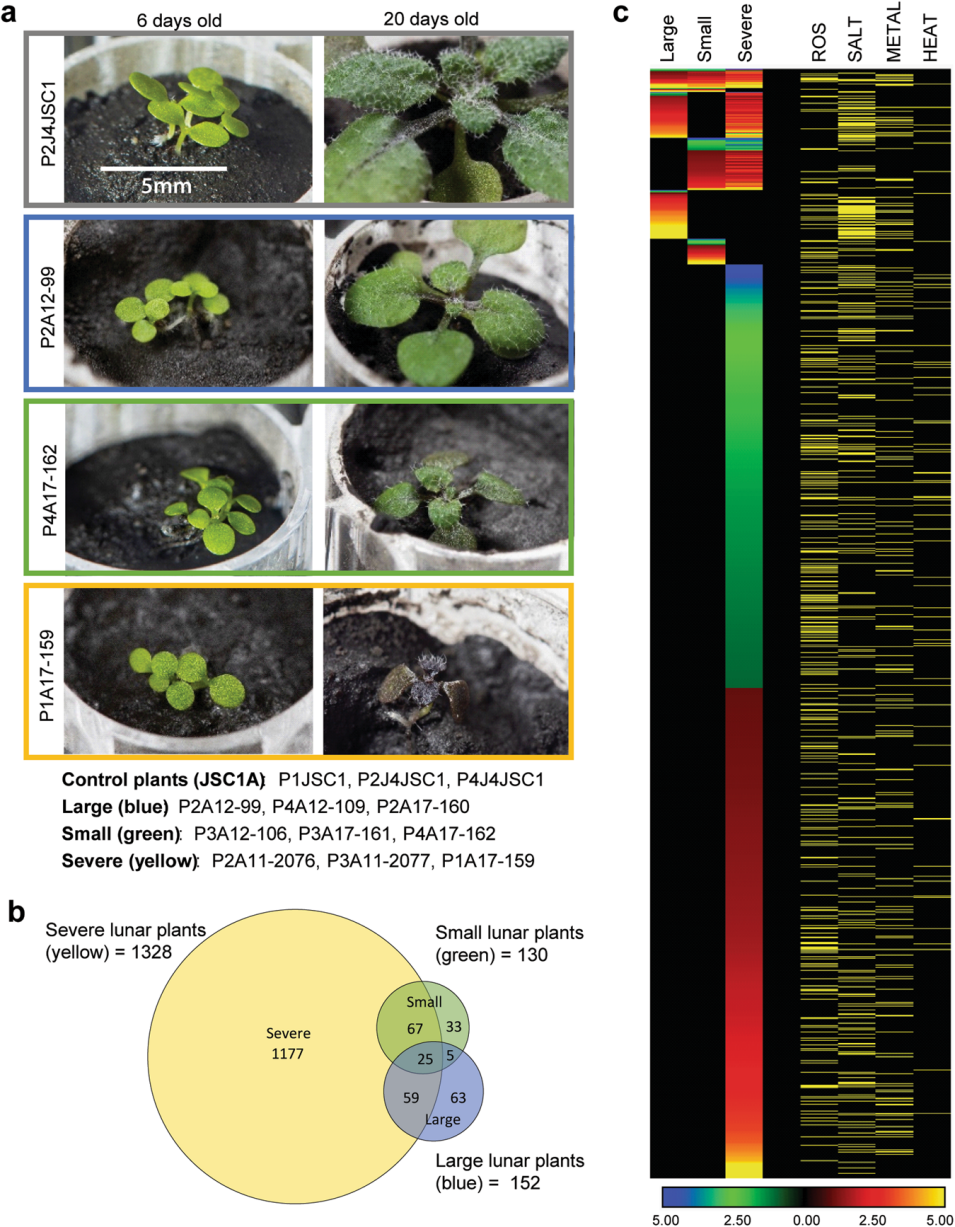
Transcriptome analyses of plants grown in lunar regolith based on morphology. **a** Representative plants from each morphology category: JSC-1A control plants (gray), large lunar plants (blue), small lunar plants (green), and severe plants (yellow). Three replicates of each type comprised each analytical set. **b** Venn diagrams showing the DEG overlap between each morphology type. **c** Heat maps show the Log2 fold-change of DEGs from each morphology type. Genes associated with ROS, salt, metal, and heat responses are indicated by yellow rows. The fully annotated heat map (4 C) is provided as Supplementary Data 2, and an annotated list of the coordinately expressed genes indicated in 4B is provided in Supplementary Data 3).

When the gene expression data were parsed with respect to relative growth success, rather than the lunar sample site, even the more successful-looking plants (those individual plants that had a size and morphology similar to those grown in JSC-1A) demonstrated strong stress-response transcriptomes (Fig. 4, Supplementary Data 2). Nine of the lunar plants were organized into three phenotypic groups of three plants each: “Severe” (tiny with distorted morphology and reddish black pigmentation throughout), “Small” (small, but green and well proportioned), and “Large” (large with respect to other regolith-grown plants, and with normal pigmentation and morphology, close to the typical JSC-1A phenotype, but still smaller than JSC-1A-grown plants). Examples of each phenotypic group are shown in Fig. 4a, and the left-hand panel also further illustrates that germination and cotyledon development is identical for JSC-1A and each of the Apollo regoliths (Fig. 3a, 4a–left panel). The quantification of the growth rates within each of these phenotypic categories is presented in Supplementary Fig. 4.

The morphologically normal Small and Large plants demonstrated only 130 and 150 DEGs compared to JSC-1A, respectively (Fig. 4b), suggesting that if plants establish a near-normal developmental trajectory at the early stages of growth, their gene expression patterns approach those of plants in JSC-1A. In contrast, the Severe phenotype plants differentially expressed well over 1000 genes, mostly stress-related, demonstrating a severe reaction to the lunar regoliths (Fig. 4b, c, Supplementary Data 2). The stochastic differentiation into these morphology groups across the three regolith sources (but not in the JSC-1A materials) suggests that plant success is at least in part driven by unique physical interactions with the lunar substrates, and the efficacious establishment of a strong root zone within the regolith. However, even the most successful lunar regolith-grown plants that overcame some of the initial physical challenges (the Large and Small phenotype categories) demonstrated stress-response transcriptomes (Fig. 4c, Supplementary Data 2). As in the site comparisons (Fig. 3), the DEGs that were coordinately expressed in all three morphology groups were predominantly associated with ROS, salt, and metal-associated stress, and most (18 of the 25) were also represented in the DEGs that were coordinately expressed in the site-specific comparisons (Figs. 3b–d, Supplementary Data 3). However, there were distinct delineations in the genes uniquely expressed in each morphology group (Fig. 4c). In the more than a thousand DEGs unique to the Severe plants, ROS-associated DEGs predominated, along with hallmarks of additional developmental stress (plant hormones, cell wall remodeling, calcium signaling; Supplementary Data 2). Of the 33 unique DEGs in the Small plants, evidence of ROS signaling also predominated, but genes associated with metal-associated stress were almost absent. In the Large, healthier-looking plants, over 60% of the 63 uniquely induced DEGs were associated with salt and drought stress, with many LEA (late embryogenesis abundant protein) family proteins being between 50 and 100-fold upregulated. The most highly down-regulated unique DEG was ATPC2 (AT1G15700), which is involved in the regulation of ATP synthase activity, which could have a substantial impact on energy metabolism (Fig. 4a; Supplementary Data 2). The DEGs of the Large, healthier-looking plants likely represent the metabolic challenges that need to be surmounted by plants in the lunar regolith, even if the physical challenges of establishing growth are met.

### Implications for lunar exploration and habitation

These data demonstrated that terrestrial plants are capable of growth in lunar regolith as the primary support matrix. Soils derived from lunar regolith could therefore be used for plant production and experiments on the Moon. However, these data also demonstrated that lunar regolith was not a benign growth substrate. Plants can fail to fully establish in the lunar regolith, resulting in a range of growth states and success. Moreover, plants broadly interpreted lunar soils as highly ionic and as eliciting oxidative stress, which is consonant with the prediction that the cosmic ray and solar wind damage to surface regolith would leave it very reactive to biological systems^44^. Fe-oxide deposits resulting from the exposure of nanophase iron to atmospheric oxygen could inhibit mineral utilization in the context of regolith^8,45^, whereas grain size shape and micropores could influence material surface area to foster an elevated release of ions into solution^46^. An increase in nanophase iron and a decrease in grain size are hallmarks of mature lunar regolith^31,47^, such as the Apollo 11 regolith used in this study (10084). Although examples of poorly developing plants were represented in each of Apollo site sources, overall, the plants grown in Apollo 11 regolith struggled the most and displayed the greatest number of differentially expressed genes. The plants grown in Apollo 17 regolith struggled the least, and displayed fewer differentially expressed genes. These data suggest that more mature regolith provides a poorer substrate for plant growth than immature regolith. Thus, although this study demonstrates that plants can use lunar regolith as a primary substrate, further characterization and optimization would be required before regolith can be considered a routine in situ resource, particularly in locations where the regolith is highly mature.

## Methods

### Plant materials

All seeds used in the experiment were from the same batch of seeds from *Arabidopsis thaliana* (Arabidopsis) ecotype Columbia-0 (Col-0) (TAIR CS70000)^48^.

### Lunar regolith materials

The lunar Allocation Analysis Review Board/AARB (formerly CAPTEM), through the Apollo Sample Curator at Johnson Space Center (Ryan Ziegler), provided 4 × 1 g samples of regolith from Apollo 11 (10084—2075, 2076, 2077, 2078), Apollo 12 (12070—105, 99,106, 109) and Apollo 17 (70051—159, 160, 161, 162) (Fig. 1a). An example of an Apollo 12 sample before opening is shown in Supplementary Fig. 1A. All lunar samples were of particle size <1 mm. These regoliths, along with the NASA lunar simulant JSC-1A (Orbitec JSC-1A Lunar, particle size <1 mm), were used as the substrate for plant growth.

### Plant growth plate configuration and habitat

Plants grew in 900 mg of material; four replicates form each site, alongside 16 replicates of JSC-1A simulant (also 900 mg each). The replicates were arranged in four, 48-well cell culture plates (Nunc 48-well sterile culture plate, cat.# 150687) such that each plate held a replicate of each Apollo site regolith (Fig. 1a, Supplementary Fig.1B), and four replicates of JSC-1A. Each well is 12.5 mm in diameter and 15 mm deep. The plates were prepared with the following steps:

1. Drainage holes were drilled in a specific configuration to distinguish groups of JSC-1A and lunar samples and then labeled (Plate 1, Plate 2, Plate 3, Plate 4) (Supplementary Fig. 1B).
2. The subsurface irrigation system was created by inserting 10 mm × 15 mm cylinders of Rockwool such that a tuft of fibers extends from the bottom of the drainage hole, which compresses the plug to about 7 mm thickness within the well. The plug of Rockwool is topped with a 13 mm circle of a nylon 0.45 μm filter to help prevent regolith from sieving into the plug (Supplementary Fig. 1C). Rockwool is a commercially available (e.g., Grodan®) material composed of spun basalt fibers compressed into formable slabs. The material is chemically and biologically inert, and widely used in plant growth applications; it could conceivably be produced from lunar basalts in situ.
3. The Rockwool plugs in the growth plates were moistened with a nutrient solution of 0.125× strength MS nutrient solution, pH 5.7 (Murashige and Skoog^49^) by placing the plates in trays of the solution to soak the plugs from below, and then allowed to drain. Next, 900 mg of each regolith sample (or JSC-1A) was added to the designated wells (Supplementary Fig. 1D), and then the plates were returned to trays of nutrient solution to wet the regolith in contact with the Rockwool plug (Supplementary Fig. 1E). The Apollo 17 samples wetted completely through capillary action (as did the JSC-1A controls), however, the Apollo 11 and 12 samples were hydrophobic and initially failed to wet using the subsurface irrigation procedure (Supplementary Fig. 1F). The Apollo 11 and 12 samples were therefore actively stirred with nutrient solution to overcome the hydrophobicity, and once wetted, the samples behaved physically similar to JSC-1A and Apollo 17 (Supplementary Fig. 1G).
4. When all samples were evenly wet with nutrient solution, Arabidopsis Col-0 seeds were distributed with a micro-pipette to the surface of each well (Supplementary Fig. 1H), and then the plates were transferred to vented terrarium chambers (Supplementary Fig. 1I) to reduce airflow yet allow air exchange with the surrounding environment.

The growth plates were moistened with nutrient solution daily by placing the plates in trays of solution until the regolith was wetted from below and then allowed to drain. The Rockwool plugs wick liquid very effectively, but also drains readily and does not retain much water. This property of Rockwool helped prevent the regolith from becoming waterlogged.

The plates of plants were photographed daily.

Between days 6 and 8, the seedlings were thinned to a single plant in each well by drawing out with forceps (Supplementary Fig. 1J). On day 20 the aerial portions of the plants (leaves and hypocotyl) were harvested by cutting at soil-level with scissors to labeled micro-centrifuge tubes (Supplementary Fig. 1J) and snap-frozen in liquid nitrogen. Samples were stored at −80°C until RNA extraction.

### Calibrations with simulants for experimental design

The above approaches and protocols were based on a set of calibration experiments conducted prior to working with lunar materials. Experiments were conducted in the 48-well culture plate form-factor with two JSC planetary simulants (Orbitec JSC-1A Lunar and JSC-Mars-1A) and commercial potting soil. All materials were sized to a collection of particles <1 mm; the JSC materials we obtained pre-sieved to that particle size fraction, and the soil was sieved in the laboratory. The plates were configured as described above with rockwool plugs and filters, and watered from below with a 0.125× MS nutrient solution. The success of this approach informed the lunar materials experiment configuration (Supplementary Fig. 3A). The concentration of the MS nutrient solution was optimized in earlier experiments using 200-well seed starting trays. Serial dilutions of 1× strength MS nutrients were compared to deionized water. A concentration of 0.125× MS supported optimal growth in a JSC-1A substrate. Plants that received water alone in the JSC-1A substrate did not develop past the first set of true leaves (Supplementary Fig. 3B). Since the nutrient composition of JSC-1A is comparable to that of the lunar regolith used in this study^50,51^ (Supplementary Data 4) it was concluded that supplementary nutrients would be necessary for successful plant growth in lunar regolith as well, but there was insufficient lunar regolith available to test this assumption.

### RNA isolation and sequencing

RNA isolation and sequencing were after the approaches described in Paul et al.^15^. RNA extraction was performed using RNeasy Plant Mini Kit (Qiagen, Hilden, Germany) according to the manufacturer’s guidelines RNA concentration was determined on Qubit® 2.0 Fluorometer, RNA quality was assessed using the Agilent 2100 Bioanalyzer (Agilent Technologies, Inc.). First, 50 ng of total RNA was used for mRNA isolation using the NEBNext Poly(A) mRNA Magnetic Isolation Module (New England Biolabs, catalog # E7490). Then followed by RNA library construction with the NEBNext Ultra II Directional RNA Library Prep Kit (New England Biolabs, catalog #E7760) according to the manufacturer’s user guide. Briefly, RNA was fragmented in NEBNext First-Strand Synthesis Buffer via incubation at 94 °C for the desired time. This step was followed by first-strand cDNA synthesis using reverse transcriptase and random hexamer primer. Synthesis of ds-cDNA was performed using the 2nd strand master mix provided in the kit, followed by end-repair and adaptor ligation. At this point, Illumina adaptors were ligated to the sample. Finally, each library (uniquely barcoded) was enriched by 12 cycles of amplification, and purified with Agencourt AMPure beads (Beckman Coulter, catalog # A63881). 48 barcoded libraries were sized on the Bioanalyzer and quantified with the Qubit® 2.0 Fluorometer. Finally, these 20 individual libraries were pooled in equimolar concentration. RNASeq libraries were constructed at the UF ICBR Gene Expression Core (https://biotech.ufl.edu/gene-expression-genotyping/, RRID:SCR_019145). Sequencing was performed at the ICBR NextGen Sequencing Core (https://biotech.ufl.edu/next-gen-dna/, RRID:SCR_019152). Normalized libraries were submitted to the “Free Adapter Blocking Reagent” protocol (FAB, Cat# 20024145) in order to minimize the presence of adaptor-dimers and index hopping rates. The library pool was diluted to 0.8 nM and sequenced on one S4 flow cell lane (2 × 150 cycles) of the Illumina NovaSeq6000. The instrument’s computer utilized the NovaSeq Control Software v1.6. Cluster and SBS consumables were v1.5. The final loading concentration of the library was 120 pM with 1% PhiX spike-in control. One lane generated 2.5–3 billion paired-end reads (~950 Gb) with an average Q30% ≥ 92.5% and Cluster PF = 85.4%. FastQ files were generated using the BCL2fastQ function in the Illumina BaseSpace portal. One NovaSeq S4, 2 × 150 cycles lane resulted in an average of 50 Million demultiplexed, paired-end reads when sequencing a pool of 48 samples.

### RNASeq bioinformatics

RNASeq Bioinformatic approaches have been previously described in Paul et al.^15^. The quality of the RNASeq sequence data was evaluated using FastQC and low-quality bases trimmed from the reads using Trimmomatic^52,53^. STAR Aligner^54^ was used to map high-quality paired-end reads to TAIR10 genome, and Gene expression values were calculated using RSEM^55^. The edgeR linear regression model^56^ was used to perform the differential gene analysis. Hierarchical clustering and principal component analysis were conducted to evaluate the association of the samples. The thresholds for calling significantly DEG were set at, FDR of 0.05, a fold change of >2, and the average FPKM (Fragments Per Kilobase of transcript per Million mapped reads) for at least one replicate of each comparison group being higher than 0. DEG lists were analyzed for overlaps using BioVenn^57^. Processing and Analysis of the RNASeq data were performed at the UF ICBR Bioinformatics Core (https://biotech.ufl.edu/bioinformatics/; RRID:SCR_019120).

### Statistics and reproducibility

The experiment compared plant growth and transcriptomes of JSC-1A lunar simulant to plants grown in regolith from three different Apollo lunar landing sites: Apollo 11, Apollo 12, and Apollo 17; JSC-1A served as the control, the three Apollo sites were the treatments. The plants were grown in sets on four replicated 48-well growth plates, each composed of four samples of JSC-1A controls (*n* = 16 total) one representative of each lunar site on each plate (*n* = 4 total). The individual plate configuration is illustrated in Fig. 1. For the transcriptome analyses, four replicates were used for the control and for each treatment (for JSC-1A, plant #4 from each plate was used) in order to have *n* = 4 controls for parity to the lunar samples. However one of the replicates in Apollo 11 did not provide viable RNA, and so for the transcriptome analyses of Apollo 11 treatment *n* = 3, while the Apollo 12 and Apollo 17 treatments *n* = 4. The statistical methods employed for the transcriptome bioinformatics are described in the Bioinformatics Methods section. The quantitative growth graphs in Supplementary Fig. 4 were derived from the daily photographs of the four replicate growth plates (plate examples in Supplementary Fig. 2. The average values of plant size were plotted along with error bars depicting the standard error of the mean. In Supplementary Fig. 4A (Grouped by Lunar Site) *n* = 4, in Supplementary Fig. 4B (Grouped by Morphology) *n* = 3. The numerical values for all points on the Supplementary Fig. 4 graphs are provided in Supplementary Data 5, such that every data point is available.

### Reporting summary

Further information on research design is available in the Nature Research Reporting Summary linked to this article.

## Supplementary information

Supplementary Information

Description of Additional Supplementary Files

Supplementary Data 1

Supplementary Data 2

Supplementary Data 3

Supplementary Data 4

Supplementary Data 5

Reporting Summary

## Data Availability

Data supporting the findings of this work are available within the paper and in the Supplementary Information files. A reporting summary for this article is available as a Supplementary Information file. The RNA-seq transcriptome data files were deposited to the Gene Expression Omnibus database (GSE188852) and the NASA GeneLab repository GLDS-476). The data sets generated and analyzed for this study are available from the corresponding author(s) upon request. The full set of daily growth images is archived in the NASA GeneLab repository (GLDS-476).
