# Supplementary material for: Plants grown in Apollo lunar regolith present stress-associated transcriptomes that inform prospects for lunar exploration
Source: Commun Biol. 2022 May 12;5:382. doi: 10.1038/s42003-022-03334-8 (PMC9098553; doi:10.1038/s42003-022-03334-8)
Supplement: Supplementary file 1 — Supplementary Information [file 42003_2022_3334_MOESM1_ESM.pdf]

Supplementary Figures - Plants grown in Apollo lunar regolith present stress-associated transcriptomes that inform prospects for lunar exploration. A-L. Paul, S.M. Elardo and R.J. Ferl

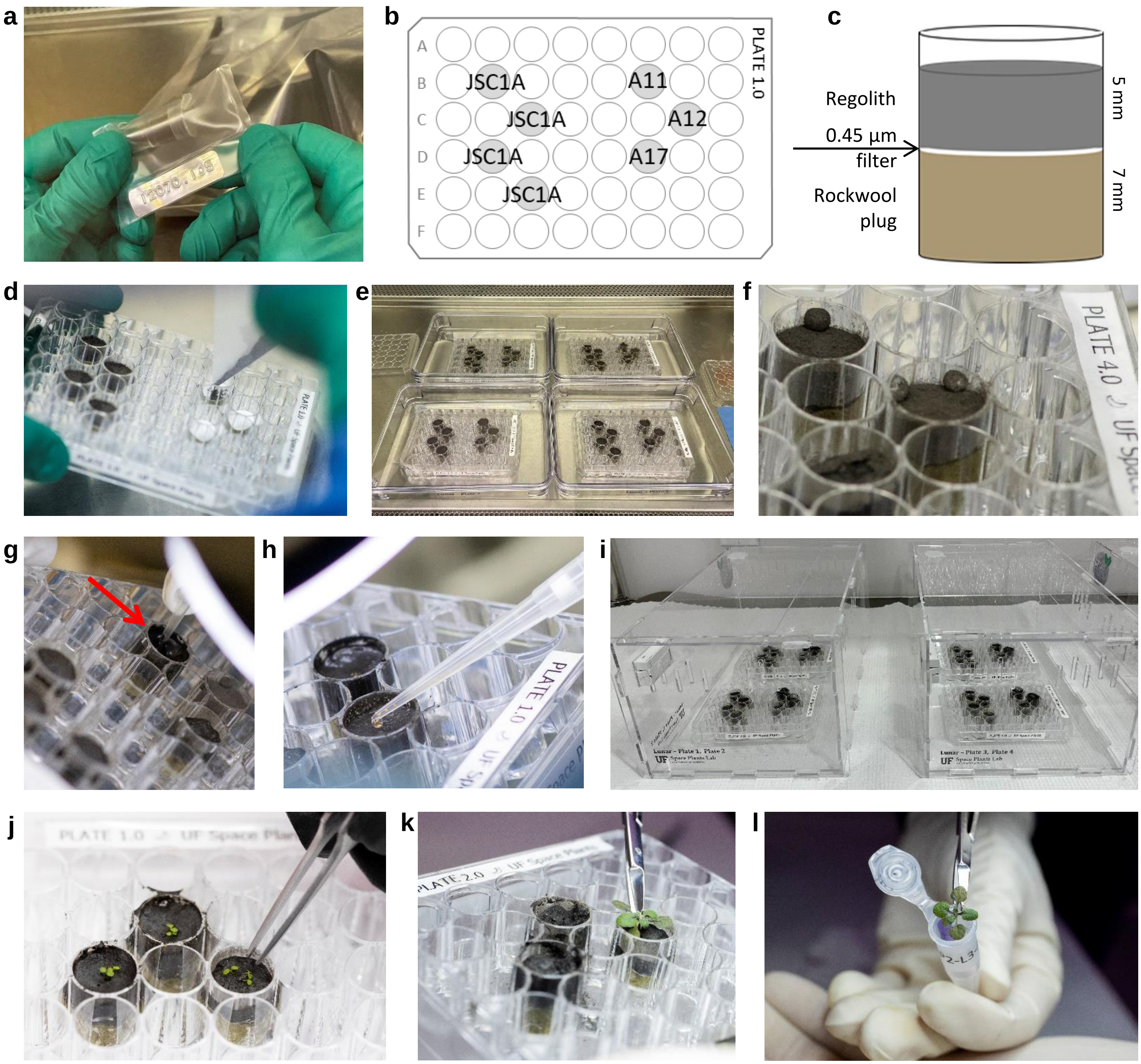

Supplementary Figure 1. A visual outline of procedures. (**a**) An Apollo 12 sample prior to opening. (**b**) A diagram of the configuration of the 48-well microtiter plate showing the well locations of the JSC-1A samples and the Apollo regolith samples. There were four replicate plates. (**c**) A diagram of the distribution of materials in a well. (**d**) 900mg of regolith was introduced into the well from the weigh paper; the 0.45µm filter that sits between the rockwool and the regolith can be seen in this photograph. (**e**) The prepared trays being soaked in nutrient solution, which revealed that the Apollo 11 and 12 samples were hydrophobic. (f) Nutrient solution dropped on the surfaced beaded and did not penetrate. (**g**) The Apollo 11 and 12 samples were actively stirred with nutrient solution to overcome the hydrophobicity. (**h**) Two to four seeds were distributed with a micro-pipette to the surface of each well. (**i**) Plates were transferred to vented terrarium chambers for growth. (**j**) Seedlings were thinned with forceps to a single plant. (**k**) On day 20 the aerial portions were harvested with scissors to labeled micro-centrifuge tubes (**l**) and snap-frozen in LN2.

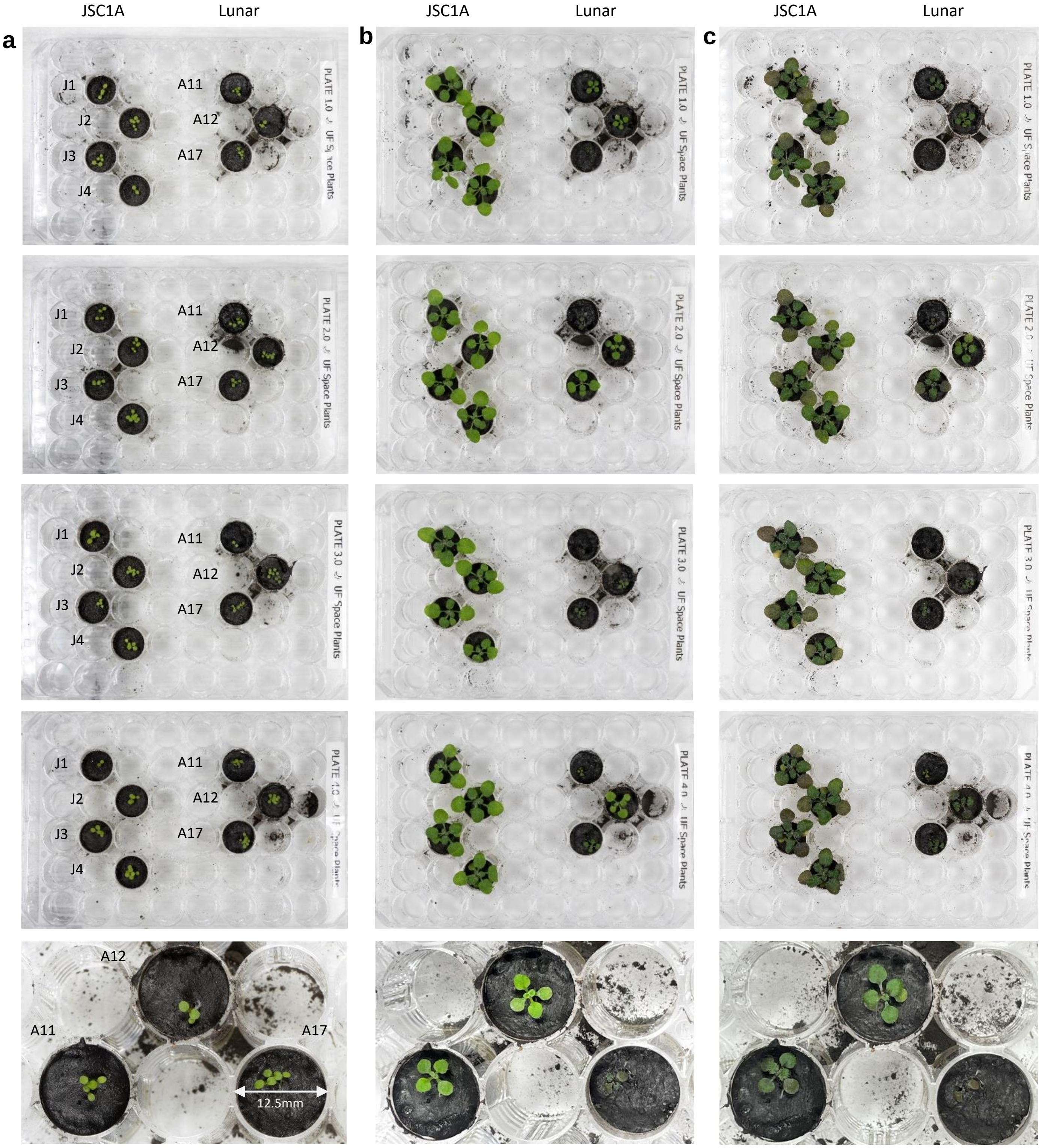

Supplementary Figure 2. Plant development in Apollo 11, 12, and 17 lunar regolith compared to JSC-1A simulant. (**a**) Plates at day 6, pre-thinning (5/4/2021). (**b**) Plates at day 16 (5/14/2021). (**c**) Plates at day 20 (harvest day – 5/18/2021). A close-up view of Apollo samples from Plate 1 are shown with each time point. The diameter of each well is 12.5 mm (white scale bar in column **a**).

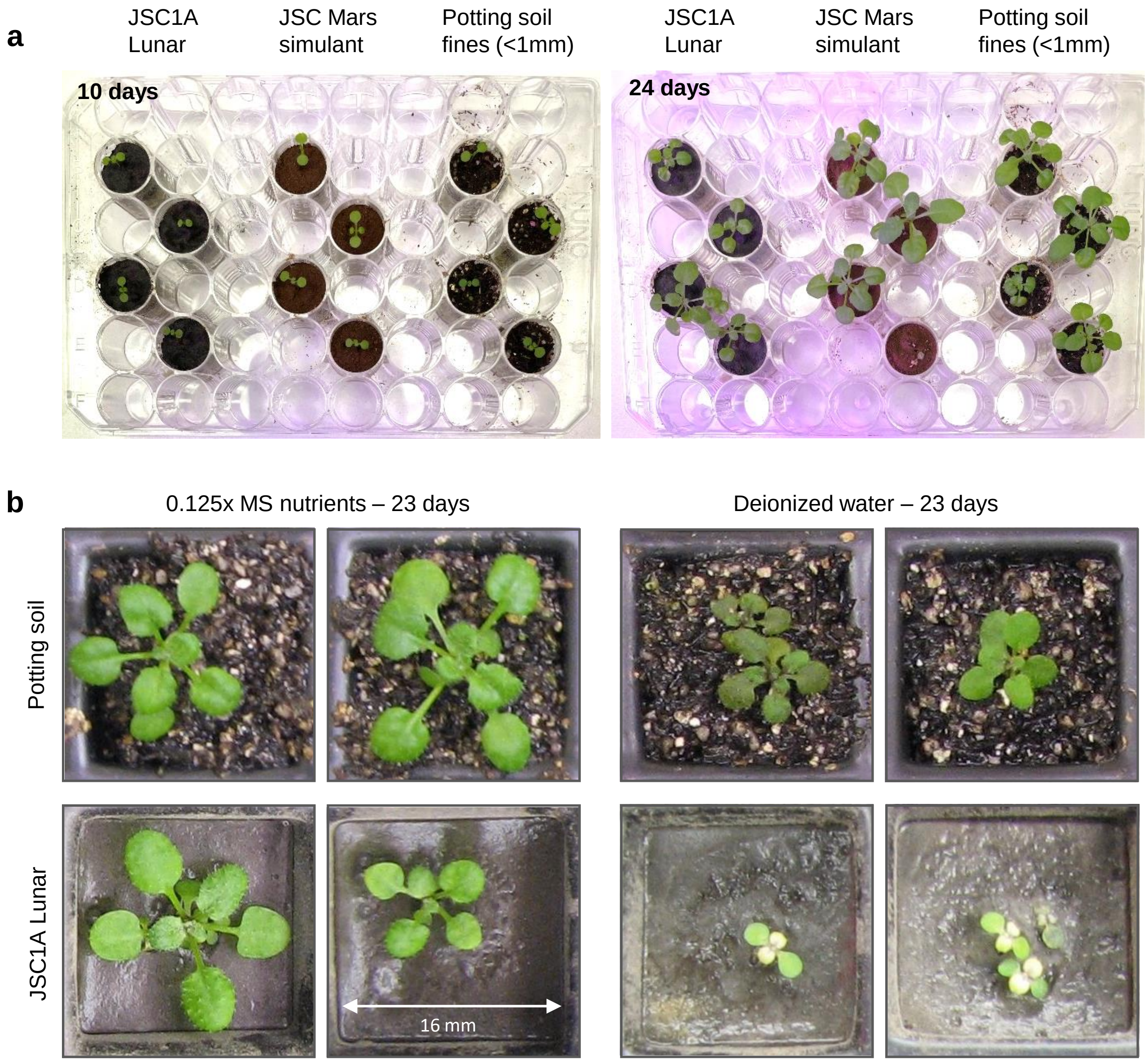

Supplementary Figure 3. Preliminary growth experiments used to define lunar regolith approaches.
(**a**) Preliminary experiments were conducted in the 48-well plates form-factor (Nunc 48-well sterile culture plate, cat.# 150687) with two JSC planetary simulants (Orbitec JSC1A Lunar and JSC-Mars-1A) and commercial potting soil. All materials were sized to a collection of particles <1mm; the JSC materials we obtained pre-sieved to that particle size fraction, and the soil was sieved in the laboratory. The plates were organized as described in Figure S1, including watering from below with a 0.125x MS nutrient solution (MS - Murashige and Skoog, A revised medium for rapid growth and bioassays with tobacco tissue culture. *Physiol. Plant.* 15, 473-479, 1962). The wells have an inner diameter of 12.5mm. (**b**) The concentration of MS nutrient solution was optimized in earlier experiments using multi-well seed starting trays with Rockwool plugs to prevent the fine substrates from sifting out of the drainage holes. The inner width of each square well is 16mm (white scale bar in **b**). Hydrating with water alone (deionized) was compared with a set of serial dilutions of 1x strength MS nutrient solution. A concentration of 0.125x MS supported optimal growth in a JSC1A substrate. Plants that received water alone in the JSC-1A substrate did not develop past the first set of true leaves, but survived in this stunted state. All plants in (**b**) are the same age and presented at the same scale.

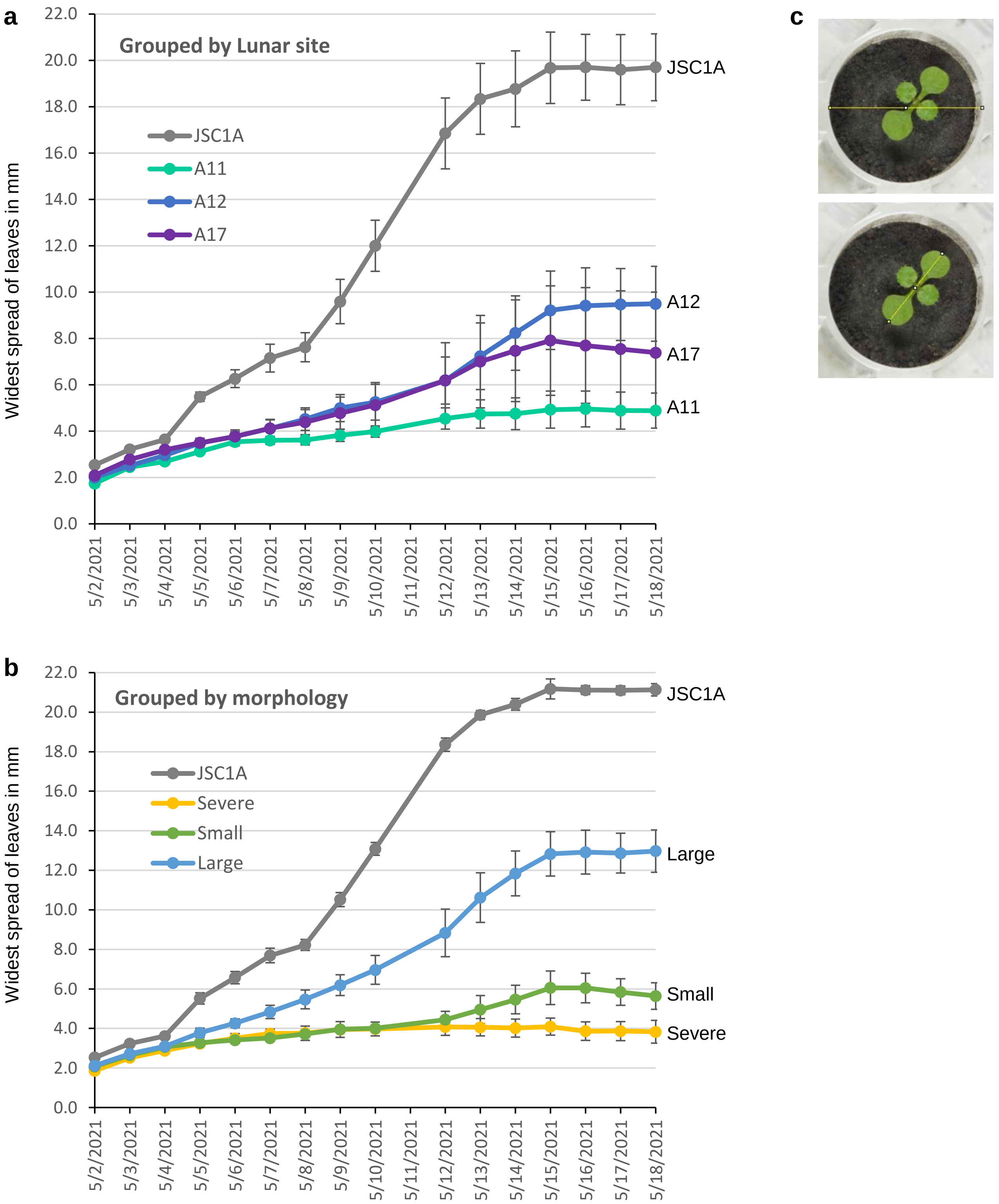

Supplementary Figure 4. Growth quantification of the plants utilized for transcriptome analyses. The canopy width of each plant that was used in the transcriptome analyses (Figure 3) was derived from the daily photographs of the four replicate growth plates (plate examples in Figure S2). The average values of plant size over time are displayed in two ways: (**a**) the replicates (n=4) from each Apollo site compared to JSC1A (n=4) (refer to Figure 3) and (**b**) the replicates (n=3) representative of each morphology group compared to three replicates of JSC1A (n=3) (refer to Figure 4). The Standard Error of the Mean (SEM) was calculated for each averaged set of replicates and the values placed as error bars in each graph. (**c**) The photo-derived values were calculated in ImageJ (Schneider et al., NIH Image to ImageJ: 25 years of image analysis. *Nature Methods* 9, 671-675, 2012) by first setting the scale with the diameter of the well, and then measuring the widest spread of the leaf canopy. The numerical values of all measurements contributing to each data point are provided in Supplementary Data 5 spreadsheet.
