# Supplementary material for: Plants grown in Apollo lunar regolith present stress-associated transcriptomes that inform prospects for lunar exploration
Source: Commun Biol. 2022 May 12;5:382. doi: 10.1038/s42003-022-03334-8 (PMC9098553; doi:10.1038/s42003-022-03334-8)
Supplement: Supplementary file 2 — Description of Additional Supplementary Files [file 42003_2022_3334_MOESM2_ESM.pdf]

Description of Additional Supplementary Files

Supplementary Data 1.

Annotations for the Figure 3 heatmap – comparisons on the basis of Apollo site.

Supplementary Data 2.

Annotations for the Figure 4 heatmap – comparisons on the basis of Apollo site.

Supplementary Data 3.

Coordinately expressed genes from Figures 3 and 4.

Supplementary Data 4.

Chemical composition of Lunar regolith used in this study alongside that of JSC-1A. Values presented as percent by weight.

Supplementary Data 5.

The numerical data for each point represented in the graphs of Figures S4a and S4b
