# Supplementary material for: Plants grown in Apollo lunar regolith present stress-associated transcriptomes that inform prospects for lunar exploration
Source: Commun Biol. 2022 May 12;5:382. doi: 10.1038/s42003-022-03334-8 (PMC9098553; doi:10.1038/s42003-022-03334-8)
Supplement: Supplementary file 8 — Reporting Summary [file 42003_2022_3334_MOESM8_ESM.pdf]

nature research

Corresponding author(s): Robert Ferl, Anna-Lisa Paul

Last updated by author(s): Feb 28, 2022

Reporting Summary

Nature Research wishes to improve the reproducibility of the work that we publish. This form provides structure for consistency and transparency in reporting. For further information on Nature Research policies, see our Editorial Policies and the Editorial Policy Checklist.

Statistics

For all statistical analyses, confirm that the following items are present in the figure legend, table legend, main text, or Methods section.

n/a	Confirmed	
☐	☒	The exact sample size (*n*) for each experimental group/condition, given as a discrete number and unit of measurement
☐	☒	A statement on whether measurements were taken from distinct samples or whether the same sample was measured repeatedly
☐	☒	The statistical test(s) used AND whether they are one- or two-sided<br>*Only common tests should be described solely by name; describe more complex techniques in the Methods section.*
☒	☐	A description of all covariates tested
☒	☐	A description of any assumptions or corrections, such as tests of normality and adjustment for multiple comparisons
☒	☐	A full description of the statistical parameters including central tendency (e.g. means) or other basic estimates (e.g. regression coefficient) AND variation (e.g. standard deviation) or associated estimates of uncertainty (e.g. confidence intervals)
☒	☐	For null hypothesis testing, the test statistic (e.g. *F*, *t*, *r*) with confidence intervals, effect sizes, degrees of freedom and *P* value noted<br>*Give P values as exact values whenever suitable.*
☒	☐	For Bayesian analysis, information on the choice of priors and Markov chain Monte Carlo settings
☒	☐	For hierarchical and complex designs, identification of the appropriate level for tests and full reporting of outcomes
☒	☐	Estimates of effect sizes (e.g. Cohen's *d*, Pearson's *r*), indicating how they were calculated

Our web collection on statistics for biologists contains articles on many of the points above.

Software and code

Policy information about availability of computer code

Data collection	software not used in data collection
Data analysis	ImageJ was used to quantify linear measurements from photographs for Supp Figure S4. Schneider, C. A., Rasband, W. S. & Eliceiri, K. W. NIH Image to ImageJ: 25 years of image analysis. Nature methods 9, 671-675, doi:10.1038/nmeth.2089 (2012)<br>RNASeq sequence data was first evaluated using FastQC. Low-quality bases were trimmed from the reads using Trimmomatic. STAR Aligner was used to map high-quality paired-end reads to TAIR10 genome.

For manuscripts utilizing custom algorithms or software that are central to the research but not yet described in published literature, software must be made available to editors and reviewers. We strongly encourage code deposition in a community repository (e.g. GitHub). See the Nature Research guidelines for submitting code & software for further information.

Data

Policy information about availability of data

All manuscripts must include a data availability statement. This statement should provide the following information, where applicable:

- Accession codes, unique identifiers, or web links for publicly available datasets
- A list of figures that have associated raw data
- A description of any restrictions on data availability

Data supporting the findings of this work are available within the paper and in the Supplementary Information files. A reporting summary for this article is available as a Supplementary Information file. The RNA-seq transcriptome data files were deposited to the Gene Expression Omnibus database (GSE188852) and the NASA GeneLab repository (GLDS-476). The datasets generated and analyzed for this study are available from the corresponding author(s) upon request. The full set of daily growth images are archived in the NASA Life Sciences Data Archive (LSDA - https://lsda.jsc.nasa.gov/)(accession through GeneLab GLDS-476).

April 2020

Field-specific reporting

Please select the one below that is the best fit for your research. If you are not sure, read the appropriate sections before making your selection.

☒ Life sciences ☐ Behavioural & social sciences ☐ Ecological, evolutionary & environmental sciences

For a reference copy of the document with all sections, see nature.com/documents/nr-reporting-summary-flat.pdf

Life sciences study design

All studies must disclose on these points even when the disclosure is negative.

Sample size	Control (plants grown on JSC-1A regolith simulant): n=16 for morphology observations and n=4 for transcriptome analyses. Treatments (plants grown on lunar regolith from thee different Apollo sites): n=4 for each of the three treatments (sites) for morphology observations and transcriptome analyses.
Data exclusions	One replicate from the transcriptomes (Treatement 1, replicate #4) as the RNA of that sample failed quality control, so for the final transcriptome analyses, the the Apollo 11 treatment site had an n=3 rather than the n=4 for all other treatments and controls
Replication	We conducted preliminary experiments as part of the initial experimental design phase, before working with bona fide lunar materials, to ensure we could grow plants in the reduced form factor (900mg material in 48-well plates) in a reliable manner that provided confidence that a replicate set of four would support the experiment. In addition, because the amount of lunar material was so small (we were only allocated 4g from each site) subdividing the material into more than 4 replicates would (based on our preliminary studies) compromised the efficacy of plant growth.
Randomization	Each replicate were grown on a separate growth plate (four, 48 well plates) to ensure that any variability associated with a given growth plate micro-habitat did not influence replicates as a group, but the nature of the experiment did not require randomization.
Blinding	Not relevant due to the small size and nature of the experiment.

Reporting for specific materials, systems and methods

We require information from authors about some types of materials, experimental systems and methods used in many studies. Here, indicate whether each material, system or method listed is relevant to your study. If you are not sure if a list item applies to your research, read the appropriate section before selecting a response.

Materials & experimental systems

n/a	Involved in the study	
☒	☐	Antibodies
☒	☐	Eukaryotic cell lines
☒	☐	Palaeontology and archaeology
☒	☐	Animals and other organisms
☒	☐	Human research participants
☒	☐	Clinical data
☒	☐	Dual use research of concern

Methods

n/a	Involved in the study	
☒	☐	ChIP-seq
☒	☐	Flow cytometry
☒	☐	MRI-based neuroimaging
